# Adaptation of the Invasive Plant (*Sphagneticola trilobata* L. Pruski) to a High Cadmium Environment by Hybridizing With Native Relatives

**DOI:** 10.3389/fpls.2022.905577

**Published:** 2022-06-28

**Authors:** Lei Gao, Minling Cai, Lingda Zeng, Qilei Zhang, Haoqiang Zhu, Xiaoqian Gu, Changlian Peng

**Affiliations:** ^1^Guangdong Provincial Key Laboratory of Biotechnology for Plant Development, Guangzhou Key Laboratory of Subtropical Biodiversity and Biomonitoring, School of Life Sciences, South China Normal University, Guangzhou, China; ^2^College of Life Science, Huizhou University, Huizhou, China

**Keywords:** *Sphagneticola trilobata*, hybrid, heterosis, cadmium, biological invasion

## Abstract

Invasive species can evolve rapidly in the invasion areas to adapt to new habitats. *Sphagneticola trilobata* L. Pruski, an invasive species, was studied for its tolerance to cadmium (Cd) in the soil and compared with its natural hybrid. From the perspective of photosynthetic physiology, antioxidant characteristics, and leaf hormone levels, the differences between the leaves of the two species before and after Cd treatment were compared. The results showed that the hybrid had stronger tolerance to Cd stress than invasive species. After Cd stress, the indexes of gas-exchange [net photosynthetic rate (Pn), intercellular CO_2_ concentration (Ci), stomatal conductance (Gs), and transpiration rate (Tr)] of the hybrid was higher than invasive species, while the content of non-enzymatic antioxidants (flavonoids and total phenols) and antioxidant enzyme activities [peroxidase (POD) and superoxide dismutase (SOD)] was lower in hybrid than in invasive species. The changes in the content of plant hormones [auxin (IAA) and abscisic acid (ABA)] under Cd stress showed that hybrid can still maintain growth and prevent leaf senescence. Furthermore, the differences in gene expression between hybrid and invasive species in photosynthetic physiology, the antioxidant capacity of leaves, and endogenous hormone (IAA and ABA) synthesis pathway also showed that hybrid has stronger Cd tolerance than invasive species. This suggests that invasive species will realize the invasion through hybridization with the native relatives to overcome the stress from environmental factors. The study implied that hybridization between invasive species and native relatives is an important way for invasive species to spread in a wider and new environment that invasive species have not experienced in the area of origin.

## Introduction

Many plant species have been introduced from their native range into new regions, where some of them have established naturalized populations and have become invasive. Therefore, the main problem with exotic plants is how to adapt the new environments in a new place. In general, exotic plants will multiply and spread rapidly in a new invaded place due to their growth and reproduction characteristics. However, studies have shown that invasive plants often occupy artificially disturbed habitats, which is very different from the native habitats of invasive plants (Skeffington and Hall, 2011; Wang et al., 2011; Heberling and Fridley, 2013). The disturbed environments often include bare land, wasteland caused by the utility of pesticides and chemical fertilizers, and abandoned land due to the free discharge of harmful substances by factories. For example, a study has shown that some abandoned lands have a high content of heavy metal Cadmium due to factory emissions (Lu et al., 2020)These habitats are easily occupied by invasive plants, which means that invasive plants may have some special adaptation strategies. Some studies suggested that invasive species can rapidly evolve to adapt to new habitats (Prentis et al., 2008; Henery et al., 2010; Oduor et al., 2016; Liu et al., 2020). However, the genetic mechanism of rapid evolution is still unclear. Many studies believed that invasive plants can adapt to the new invasive environment through a rapid evolution from the perspective of morphology and physiology, such as the study by Feng et al. (2009) showed that invasive species increased the utilization efficiency of nitrogen in the cytoplasm and reduced the nitrogen content in the cell wall so that invasive species can use more nutrients for growth and reproduction. In spite of this, the rapid evolution of invasive species still needs to be further analyzed from the gene level (Gruntman et al., 2020). With the development of molecular biology, the mechanism of plant invasion has been revealed more from the level of heredity and gene. The study showed that the invasive plant *Solidago canadensis* L. has stronger adaptability after chromosome doubling and can spread to the low latitude zone with higher temperatures in the invasion area (Cheng et al., 2021).

Many exotic species in newly invaded areas will produce hybrid crossbreeding with the same genus species or sibling species of the native area (Gao et al., 2018). Due to the hybrid inheriting the native and original parents’ characteristics, the reproduction ability, adaptability to the environment, etc., often appear better than the parents’ ability, thus becoming the most important ways of biological invasion of exotic species, such as *Polygonum caespitosum* Bl., *Salix caprea* L., *Gomphocarpus physocarpus* E. Mey., *Psidium guajava* L., *Stenotaphrum secundatum* (Walt.) Kuntze, and *Stachytarpheta urticifolia* (Salisb.) Sims (Gao et al., 2018). In this way, cross-fertilization may be an important process in invasion biology where genotypes from various origins are introduced into a new area, so, genetic hybrid processes may also play important roles (Bleeker and Matthies, 2005; Groeneveld et al., 2014; Huska et al., 2016). However, most of the evidence in this aspect is limited to ecological studies, lacking more direct evidence in molecular genetics.

In this study, *Sphagneticola trilobata* (L.) Pruski (Asteraceae) was taken as an example to explore the adaptability of its hybrid to environmental factors during the invasion. *S. trilobata*, native to South America, is an annual herbaceous liana. The plant often grows on shores or marshes, but it also has a certain degree of drought tolerance. In China, the plant is widely invasive in Guangdong, Guangxi, Fujian, and Hainan. In these regions, there is a native plant *Sphagneticola calendulacea* (L.) Pruski belonging to the same genus as *S. trilobata*. But the habitats of these two species are different. Our previous study has identified that *S. trilobata* is more resistant to water than *S. calendulacea* (Zhang et al., 2020a). In the natural environment, studies have reported that there are hybrid species, of these two species, *S. trilobata* is paternal and *S. calendulacea* is maternal (Wu et al., 2013; Li et al., 2015). In our previous research, we found that the tolerance of hybrid to water stress was in the middle of parents (Zhang et al., 2020a). For *S. trilobata*, the adaptability to water stress of hybrid enlarged the invasion and propagation of the genes of *S. trilobata* in the invasive region. Therefore, we believe that, in addition to the water factor, the hybrid may have wider adaptability to other environmental factors than the *S. trilobata*, because the hybrid combines some genotypes from parents and thus shows hybridization advantages (Zhang et al., 2020a).

We select the heavy metal Cadmium (Cd) as the environmental factor and try to study the tolerance of *S. trilobata* and its hybrid to Cd ion concentration in soil. The increase of Cd in the soil is related to human interference with the natural environment, and this aspect has become an important indicator of soil and water environmental pollution (Zhang et al., 2020a). The study has found that *S. trilobata* is the dominant species in the wasteland of Cd-rich lead–zinc mining areas in Guangdong, China (Xiao et al., 2021). Cd in the soil is easily absorbed by plants, therefore, some studies have tried to use *S. trilobata* to absorb Cd and bioremediate Cd contaminated soil (Lu et al., 2020).

*S. trilobata* mainly grows near marshes or damp places such as banks of the river, and these places are often the most serious places of human disturbance (Cai et al., 2020; Sun et al., 2020; Zhang et al., 2020a). Because of pollution and waste emissions, soil Cd ions are often very high. Therefore, the study of the adaptability of *S. trilobata* and its hybrid to the high Cd soil environment will further reveal the invasion in the genetic strategy in adapting to the environment of the invasion area. This will provide strong evidence to explain the rapid invasion and gene diffusion of invasive species.

Plants growing in high Cd soil will show a series of symptoms in phenotypes, and plant physiological processes. At high Cd stress, Cd will restrain the synthesis of chlorophyll, reduce plant photosynthetic productivity, and interfere with the transport and distribution of plant nutrients, and then, the plant will grow slowly and become short, green back, production decline, and so on (da Silva et al., 2012; Hu et al., 2014; Lomaglio et al., 2015). At the cellular level, high levels of Cd in plants can interfere with the cell cycle, interfere with DNA synthesis, change cell membrane permeability, and change the activity of enzymes in cellular physiological and biochemical processes, thus interfering with the whole cellular physiological and biochemical process (Surgun-Acar, 2018).

Even so, in order to maintain the tolerance range of Cd ion concentration in the plants, the plants have a steady-state regulation mechanism of heavy metals, such as root secretion, cell wall fixed effect, the selective permeability of plasmalemma, the roles of vacuole isolation, and chelating effect at the molecular level, etc. These ways can control the absorption, transport, and accumulation of Cd ion in the plant to minimize the damage of Cd. Studies have shown that plants can accumulate a large amount of reactive oxygen species under stress. In order to avoid oxidative damage, plants can activate an antioxidant system, including enzymatic antioxidants and non-enzymatic antioxidants. This system can remove excessive reactive oxygen and maintain a low level of reactive oxygen (Finger-Teixeira et al., 2021; Pardo-Hernandez et al., 2021; Zhang et al., 2021). In addition, under a Cd stress environment, the levels of plant hormones, including auxin (IAA), abscisic acid (ABA), cytokinin (CTK), gibberellic acid (GA), etc., will be affected under high Cd level. Therefore, the change level of plant hormones can also indicate the tolerance to Cd (El Rasafi et al., 2020).

Based on this, we studied the invasion strategies of *S. trilobata* and its hybrid. From the level of photosynthetic physiology, enzyme activity and related hormone levels, and transcriptome level, we studied the tolerance of these two plants to Cd stress, and then discussed the significance of the hybrid strategy of invasive species with native species in the process of plant invasion. We tried to prove whether the hybrid is more resistant to Cd stress than invasive species, thus we can prove that hybridization with native species is one of the important ways for plant invasion success to achieve gene transmission of invasive species. Here, we want to put forward the hypothesis: alien species produce invasive species through hybridization with native relatives. This will be a strategy for invasive species to quickly adapt to the invasion environment. This strategy makes the genes of alien species can be inherited and transmitted in more stressful environments through hybrids.

## Materials and Methods

### Materials

*Sphagneticola trilobata* mainly propagates asexually and its sexual ability is unusual. In the field, researchers identified a new companion species with a phenotype between the *S. trilobata* and *S. calendulacea* and suspected to be a hybrid of these two species (Wu et al., 2013). The researchers then measured the plant physiological and ecological indicators of the hybrid species. A series of photosynthetic physiological indicators and nitrogen utilization efficiency of the hybrid species were in between *S. trilobata* and *S. calendulacea*, and then, it was confirmed that the new species was indeed a cross of two plants by analysis of microsatellite and nrITS data sequences (Wu et al., 2013; Li et al., 2015; Sun et al., 2015). The hybrid is a natural hybrid, therefore, it has been planted and reserved in the South China Botanical Garden, Chinese Academy of Sciences under strict anti-diffusion conditions.

We selected invasive species and the hybrid as experimental materials. All the materials were collected from the South China Botanical Garden. The Garden is located in Guangzhou city, and the latitude and longitude are 23° 10 ′ N, 113° 21′ E. We trimmed the collected plant materials into small segments with two internodes 8-10cm long and placed them in a culture bottle for cutting propagation. After 2 weeks of low-light culture, regenerated seedlings with a similar number of leaves were selected for the Cd stress experiment, 1 plant per bottle (20 cm height, 10 cm diameter).

### Treatment Setting and Parameter Measurements

After growing out 3–4 pairs of leaves, the plants of two species were selected for the Cd tolerance experiment. Each species was divided into a control group and a treatment group. For the treatment group, CaCl_2_ with a concentration of 200 μmol/L was used as the Cd tolerance reagent, while the control group did not add Cd. In the experiment, Hogland’s nutrient solution was added to all plants as a nutrient. All plants were set as repeats for each group of plants. We observed the changes in the phenotype of two species with the days of Cd stress. After 2 weeks, two species were tested for their tolerance to Cd stress. The parameters include (1) gas-exchange: net photosynthetic rate Pn, intercellular CO_2_ concentration Ci, stomatal conductance Gs, and transpiration rate Tr; (2) antioxidant enzyme activities (Peroxidase POD and superoxide dismutase SOD); (3) content of plant hormones (IAA and ABA); and (4) RNA transcriptome analysis of leaves of two plants.

For the parameters of gas exchange, an infrared gas analyzer Li-6400 (LI-COR, Inc., United States) was used to measure leaf gas exchange. We choose the third leaf from the top of each plant for measuring materials. During the measurements, reference CO_2_ concentration in the leaf chamber was maintained at ca. 500 μmol/mol. To ensure that plants were in a better photosynthetic state, the measurements were performed in the morning (08:30–12:00) and leaves were fully adapted for ∼10 min to a saturating light intensity [1,000 μmol (photon)/m/s] provided by an LED light source. The net photosynthetic rate (Pn), intercellular CO_2_ concentration (Ci), stomatal conductance (Gs), and transpiration rate (Tr) were automatically recorded and stored by the computer systems of the device.

Peroxidase (POD) activity was determined with guaiacol by spectrophotometry (Lagrimini, 1991). In the presence of H_2_O_2_, POD catalyzes the transformation of guaiacol to tetraguaiacol. This reaction can be recorded at 470 nm. The reaction mixture contained 100mm phosphate buffer (pH 6.0), 33 mm guaiacol, and 0.3 mm H_2_O_2_. The enzyme-specific activity was expressed as ΔOD470/min/g FW (OD: optical density).

Superoxide dismutase (SOD) was assayed on the basis of its ability to inhibit the photochemical reduction of nitro blue tetrazolium (Beauchamp and Fridovich, 1971). The reaction mixture contained 50 mM phosphate buffer (pH 7.8), 13 mM methionine, 75 μm nitro blue tetrazolium, 2 μm riboflavin, 100 nM EDTA, and 0–200 μl of enzyme extract. The riboflavin was added last. The reaction mixture was read at 560 nm. One unit of SOD activity (U) was defined as the amount of enzyme that caused 50% inhibition of the initial rate of the reaction in the absence of enzyme. Total SOD activity was expressed as U/min^/^gFW.

We also carried the localization of reactive oxygen species (ROS) in plant leaves. At first, five fresh leaves of each group including Cd stress and control were collected, then all the leaves were quickly dipped into phosphate buffer (pH1.0) containing 2-aminobenzidine (0.5 mg/ml) to ensure that all leaves were immersed in the solution. Rapid vacuum for 10 min then placed the leaves in the dark for 8h. When the leaves appear with brown spots, take out the leaves and boil them in a solution of ethanol (95%) until the leaves turn yellow-white. Finally, the leaves were observed under a stereoscope and photographed.

In addition, we also measured the changes in plant biomass with the days of the Cd-tolerant experiment (35days). Because it was hydroponics, we took out the plants and weighed the fresh weight when no more water was dripping. The fresh weight was taken as the biomass of the plant. After weighing, put the plant back into the bottle for culture to continue.

### RNA-Seq Analysis

Total RNA of leaf samples of each species was isolated using RNA prep Pure Plant Kit [Sangon Biotech (Shanghai) Co., Ltd., China] and Hipure total RNA Mini Kit (Guangzhou Magen Biotechnology Co., Ltd., China). The samples of RNA were tested by UV spectrophotometer, and the samples with poor quality were re-extracted.

Each qualified total RNA sample of two species was digested with DNase I, and the mRNA was enriched with magnetic beads with Oligo (DT), and then the mRNA was broken into short fragments in a thermomixer at an appropriate temperature by adding fragmentation buffer. Using mRNA as a template, one strand of cDNA was synthesized by random primer method, and then synthesize a double strand of cDNA was in a two-strand synthesis reaction system. The double-stranded cDNA was subjected to terminal repair, purification, and recovery using a kit, and was added the base A at the 3′ end of the cDNA, and then the sequencing connector was connected. Then, 2% agarose gel electrophoresis was used to select the size of the fragments, and the cDNA fragments with suitable molecular weight were recovered and amplified by a PCR kit. The constructed library was tested by using the Agilent 2100 Bioanalyzer and ABI Step One Plus Real-time PCR system. After passing the test, it was sequenced by Illumina Hiseq 4000 platform.

The raw reads obtained by transcriptome sequencing are filtered to remove the sequences with low quality, joint pollution or high content of unknown base N. The obtained clean reads were spliced by *de novo* using Bridger software to obtain the Unigene sequences of each sample, and then the function of Unigenes was annotated. The obtained Unigenes were used as the reference genes for subsequent analysis to test the differentially expressed genes between different samples.

The Unigene sequences of two species were blast compared with the databases of Non-redundant protein (NR), Non-redundant nucleoside (NT), Swiss-prot, Kyoto Encyclopedia of genes and genes (KEGG), cluster of orthologous groups of proteins (COG), and Gene Ontology (GO) for functional annotation and functional classification information.

The clean reads of leaves of two species before and after Cd treatment were compared and annotated with their Unigene library, and the gene expression level was analyzed. The expression amount of Unigene was calculated by the DESeq2 method, and the multiple changes [log2ratio (treatment/control)] of all unigenes of the two species were calculated, and then the differentially expressed genes were screened. The probability of differential expression between the treatment group and the control group was >0.05 and the absolute value of log2ratio > 1 was used as the screening conditions of differentially expressed genes. The differentially expressed genes were enriched by GO and KEGG.

### Data Analysis

One-way ANOVA (Cd treatment as a factor) was used to compare the differences in biomass, photosynthesis (Pn, Ci, Gs, Tr), plant hormones (IAA, ABA), and antioxidant indices (POD, SOD) between invasive and hybrid species before and after Cd treatment. *Post hoc* comparisons between Cd treatment and control were conducted with Tukey’s HSD test. The results are presented as non-transformed means. The significance values in the text are at *p* < 0.05 unless stated otherwise.

## Results

**FIGURE 1:**
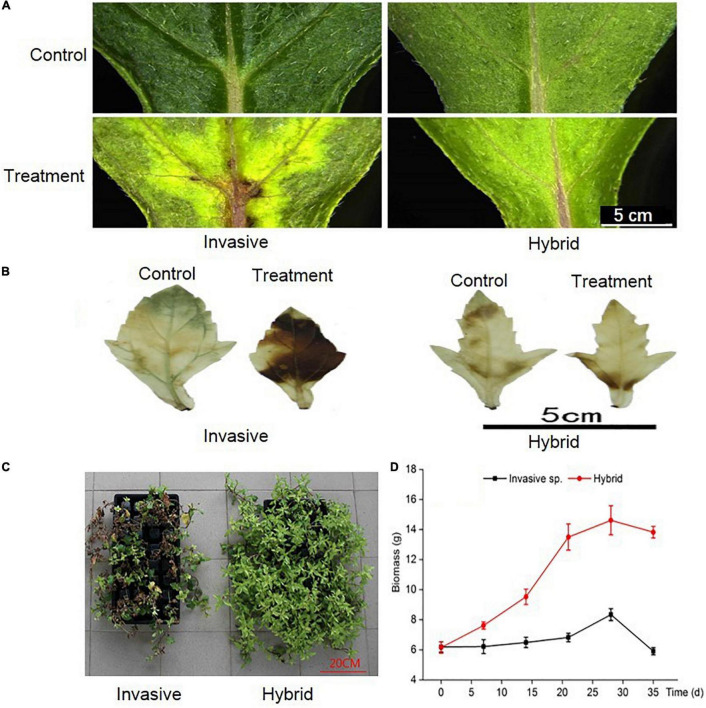
Under Cd stress, the changes of two species in phenotypic of leaves **(A)**, location of reactive oxygen in leaves **(B)**, phenotypic of whole plant **(C)**, and biomass of whole plant **(D)**.

After 14 days of dealing with Cd stress, the leaves turn yellow, and some necrotic plaques appeared at the base of leaves, esp. in invasive species, the leaves changed more yellow than a hybrid (Figures 1A,C). Under Cd stress, the plants will accumulate H_2_O_2_, but the accumulation degree of H_2_O_2_ in the two species is different. By comparing the location of H_2_O_2_ in the leaves before and after Cd stress treatment, it was found that the accumulation degree of H_2_O_2_ in the leaves of hybrid species was significantly lower than that of invasive species, only at the base of the leaves, but in the invasive species, H_2_O_2_ was accumulated in most part of the leaf (Figure 1B). In addition, after Cd stress treatment, we harvested all the plants, and the results showed that the biomass of the hybrid was higher than the invasive species (Figure 1D). So, compared with hybrid species, the Cd tolerance of invasive species was weak.

### Impact of Cd Stress on Biomass, Photosynthesis, Antioxidant Enzymes, and Hormones

**FIGURE 2:**
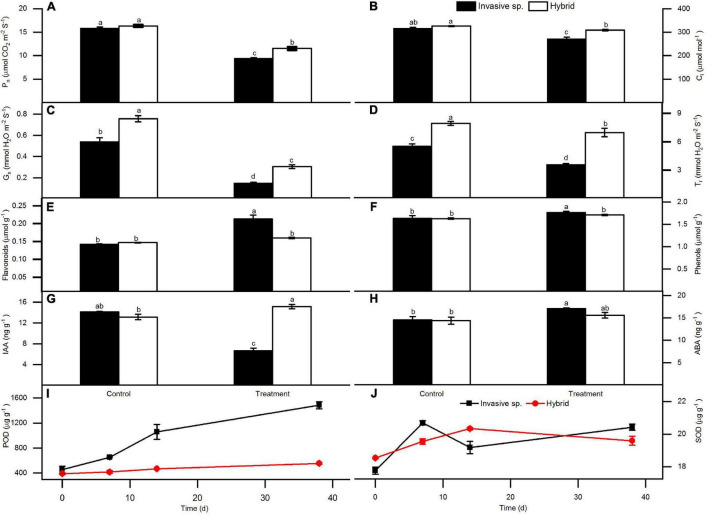
Under Cd stress, the changes of two species in photosynthetic indexes of leaves: Net photosynthetic rate (Pn) **(A)**, intercellular CO_2_ concentration (Ci) **(B)**, stomatal conductance (Gs) **(C)**, and transpiration rate (Tr) **(D)**; non-enzymatic antioxidant content: flavonoids **(E)**, total phenols **(F)**; hormone contents of plant endogenous auxin IAA **(G)** and abscisic acid ABA **(H)**; enzymatic antioxidant content: peroxidase POD **(I)** and superoxide dismutase SOD **(J)**, show Mean ± SE, One-way ANOVA, and the letters on the column indicate the difference.

Statistics showed that the hybrid was higher than invasive species in all the photosynthetic indices (Pn, Ci, Gs, Tr) under Cd stress (Figures 2A–D). After Cd treatment, the content of non-enzymatic antioxidants (flavonoids and total phenols) in leaves of invasive species increased significantly. However, the hybrid did not increase significantly comparing the Cd control (Figures 2E,F).

The content of enzymatic antioxidant enzymes (POD and SOD) in the leaves of the two plants increased along with the time of Cd treatment (Figures 2I,J). But, in invasive species, these two indices were significantly higher than in hybrid, especially to POD, the content difference between these two plants is very obvious. In addition, the analysis of non-enzymatic antioxidant flavonoids and total phenols in leaves also showed a similar pattern. It also showed that the increase of these two substances in invasive species was more significant under Cd stress, while in hybrid, these two substances did not increase significantly.

Analysis of plant hormones in leaves showed that the content of IAA in the leaves of invasive species decreased significantly under Cd stress, while in the leaves of hybrid, the content of IAA increased significantly compared to Cd control (Figure 2G). In addition, the analysis of the content of ABA in leaves further indicated that under Cd stress, it was increased significantly in invasive species, although in the hybrid it is also increased, the content was far lower than that of invasive species (Figure 2H).

### Impact of Cd Stress on the Expression of Related Genes in Photosynthesis Pathway, an Antioxidant Enzyme, and Hormone Signal Transduction

We extracted the total RNA of leaves of two species before and after Cd treatment (12 leaf samples), and then, the cDNA library was constructed by Novogene Co., Ltd., Beijing, China. Then, the purified libraries were sequenced using Illumina HiSeq™ 2500 (San Diego, CA, United States). The sequence data of each species were combined as a reference genome (Table 1). After *de novo* and redundancy, we used Busco to evaluate the assembly integrity.

**TABLE 1:** De NOVA results of leaves of two species after Cd stress treatment.

Species	*S. trilobata*	Hybrid
Transcripts	302,998	338,520
Unigenes	227,843	259,043
C values	83.7%	81.6%

For annotations of new genes, the unigenes of two species were aligned to the publicly available protein databases, including Nr, Nt, Swiss-Prot, KOG, KO, GO, and PFAM, using the BLASTx algorithm with a cut-off E-value of 10^–5^ (Table 2).

**TABLE 2:** Functional annotation of unigenes from two species.

Annotated-database	*S. trilobata*		Hybrid	
		
	Amount	Percent	Amount	Percent
Total unigenes	227,843	100	259,043	100
Nr-Annotated	99,631	43.72	110,159	42.52
Nt-Annotated	57,211	25.1	59,946	23.14
KO-Annotated	39,446	17.31	43,327	16.72
GO-Annotated	72,674	31.89	77,148	29.78
KOG-Annotated	23,208	10.18	24,106	9.3
PFAM-Annotated	71,368	31.32	75,807	29.26
Swissprot-Annotated	77,477	34	83,327	32.16

After comparing the numbers of transcriptome genes of two species, we found that, under Cd stress, the number of differential expression genes in invasive species was higher than in the hybrid (Supplementaty Table 1), which suggested that the physiological processes of invasive species have changed significantly under Cd stress.

**FIGURE 3:**
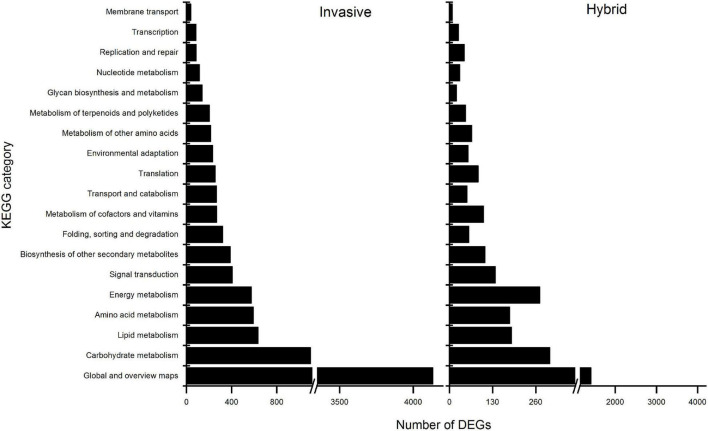
Under Cd stress, KEGG annotation of DEGs in the leaves of invasive plants and hybrid.

Furthermore, the enrichment analysis of biological pathways by KEGG to DEGs showed that all the DEGs of the two species were enriched on 20 categories of pathways (Figure 3). Among these, in invasive species, the differential expressed genes were enriched in two metabolic pathways, including Plant hormone signal transduction, photosynthesis, and flavonoid biosynthesis pathways; while in the hybrid, the genes were significantly enriched in plant hormone signal transduction and Photosynthesis pathways, and not in flavonoid biosynthesis pathway. Thus, it was shown that in the hybrid, the impaction of Cd stress was less than invasive species in the expression of genes-related flavonoid biosynthesis pathway.

### Expression of Photosynthesis-Related Genes in Two Species Under Cd Stress

The transcriptome data of photosynthesis showed that the expressions of genes associated with the photosynthetic pathway have changed under Cd stress, and the pathways of photosynthetic include Photosystem I, Photosystem II, Cytochrome b6/f complex, electron transport carrier, and ATP synthase (Table 3). In these pathways, the expression of key protein genes was generally down-regulated, such as photosystem II *CP43* reaction center protein (*PsbC*) and oxygen-evolving enhancer protein (*PsbP*) in Photosystem II, Cytochrome c1(*PetC*) gene and plastocyanin (*PetE*) gene in photosynthetic electron transport carrier, γ and β genes of ATP synthase. We analyzed the differences in the number of genes that respectively enriched in the pathway of photosynthesis of two species. It was found that compared with invasive species, the number of downregulated genes of a key protein in the photosynthetic pathway was fewer in hybrid (Table 4). This pattern was similar to the difference between the two species. Therefore, the downregulation of photosynthesis-related genes further confirmed that invasive species were not tolerant to Cd stress, and the hybrid was more tolerant to Cd stress.

**TABLE 3:** The expression multiples of key genes related photosynthetic, flavonoid synthesis, abscisic acid ABA and auxin IAA synthesis pathways in invasive species and hybrid under Cd stress.

Gene(Protein)	Hybrid	Invasive
**PhotosyntemII**		
Photosystem II CP43 reaction center protein (*PsbC*)	0	–3.3
Photosystem II CP47 reaction center protein (*PsbB*)	0	2.3
PSII reaction center subunit V (*PsbE*)	–1.3	0
Oxygen-evolving enhancer protein 2 (*PsbP*)	–6.6	–6.5
Oxygen-evolving enhancer protein 1 (*PsbO*)	–7.6	–7.2
Oxygen-evolving enhancer protein 3 (*PsbQ*)	–6.4	–6.5
Photosystem II 10 kDa polypeptide (*PsbR*)	–6.2	–6.0
Photosystem II protein Y (*PsbY*)	–1.7	–5.1
Photosystem II 11 kDa protein (*Psb27*)	–2.2	–3.8
Photosystem II 13 kDa protein (*Psb28*)	–1.1	–2.5
**PhotosyntemI**		
Photosystem I reaction center subunit K (*PsaK*)	–5.6	–10.6
Photosystem I reaction center subunit N (*PsaN*)	–5.0	–6.9
Photosystem I reaction center subunit O (*PsaO*)	–1.6	–5.0
**Cytochrome b6/f complex**		
Cytochrome f (*PetA*)	0	–2.8
Cytochrome c1 (*PetC*)	–6.0	–6.2
**Electron transport**		
Plastocyanin (*PetE*)	–5.3	–5.7
Cytochrome c553 (*PetJ*)	0	–2.4
**ATP synthase**		
ATP synthase subunit gamma (γ)	0	–2.1
ATP synthase subunit alpha (α)	–1.7	–6.5
ATP synthase subunit delta (δ)	–1.7	–2.0
**Flavonoid synthesis**		
Chalcone synthase (*CHS*)	2.9	5
Chalcone isomerase (*CHI*)	1.1	6.7
Flavanone-3-hydroxylase (*F3H*)	0	1.5
Flavanone-3-hydroxylase (*DFR*)	4.1	0
Transcriptional activator protein (*ANR*)	0	0
Anthocyanidin synthase (*ANS*)	6	1.5
Pcoumarate 3-hydroxylase (*C3H*)	1.7	5
**ABA synthesis**		
Pyrabactin resistance/pyr1-like (*PYR/PYL*)	2.1	1.1
Serine/threonine protein phosphatase (*PP2C*)	2.8	1
Sucrose non-fermenting 1-related protein kinase 2 (*SnRK2*)	1.3	2
ABA responsive element binding factors (*ABFs*)	2.3	1.5
**IAA synthesis**		
Auxin	0	–1.6
Auxin receptor (*TIR1*)	0	–1
Auxin/indole-3-acetic acid (*Aux/IAA*)	3.7	–1.4
Auxin response factor (*ARF*)	4.9	–1.4
Gretchen hagen 3 (*GH3*)	0	0
Small auxin-up RNA (*SAUR*)	1.1	–1.6

**TABLE 4:** Under Cd stress, the up and down regulated percentage of synthesis and signal regulation genes in photosynthesis, secondary metabolites, and hormones in invasive and hybrid species.

	Hybrid		Invasive	
		
Pathway	Up%	Down%	Up%	Down%
Photosynthesis	0	23.2	2.6	44.6
Flavonoid biosynthesis	9.5	0	32.0	3.3
Hormone signal transduction	4.6	2.2	13.2	10.1
IAA signal transduction	1.7	1.1	8.9	18.5
ABA signal transduction	6.4	0.5	19.3	3.4

Under the Cd stress, the key protein genes related to the photosynthesis pathway of invasive species were downregulated, such as photosystem I, photosystem II, cytochrome b6/f complex, electron transport vector, and key protein genes of ATP synthase, but the effects of Cd stress on the hybrid were not obvious in the photosynthetic process (Table 3). The biomass of the hybrid was higher than invasive species under Cd stress. This indicated that the photosynthetic process of invasive species was inhibited by Cd stress, while the hybrid can maintain higher photosynthetic capacity under the Cd stress environment.

### Expression of Antioxidant Enzyme-Related Genes in Two Species Under Cd Stress

**FIGURE 4:**
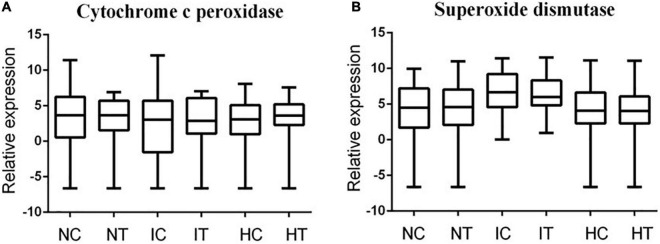
Under Cd stress, expression of regulatory genes related to the enzymatic antioxidant pathway in leaves of two species, **(A)** expression of peroxidase gene, **(B)** expression of superoxide dismutase gene.

Transcriptome analysis showed that Cytochrome C peroxidase, a gene related to POD regulation, was upregulated under Cd stress, and the most upregulation was in invasive species, while the hybrid was lower than invasive species (Figure 4A). On the contrary, the genes related to SOD regulation were downregulated significantly in invasive species, but not in the hybrid (Figure 4B). The significant upregulation of the genes coincided with the significant increase of POD content in invasive species.

**FIGURE 5:**
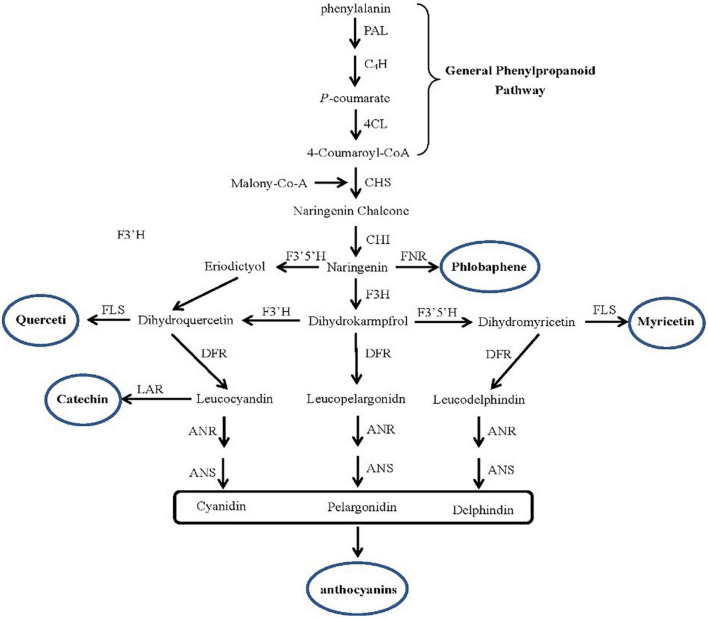
The key genes in the flavonoid synthesis pathway. The DEGs include Chalcone synthase (*CHS*), Chalcone isomerase (*CHI*), Flavanone-3-hydroxylase (*F3H*), Flavanone-3-hydroxylase (*DFR*), Transcriptional activator protein (*ANR*), Anthocyanidin synthase (*ANS*), Coumarate-3-hydroxylase (*C3H*) in invasive plant and hybrid under Cd stress were located in the pathway.

In addition, under Cd stress, plants not only activate the response of enzymatic antioxidant enzymes but also activate non-enzymatic antioxidant systems, such as flavonoids, total phenols, and anthocyanins. The expression profile analysis of key genes in the flavonoid synthesis pathway (Figure 5) showed that the genes were upregulated in two species, such as chalcone synthase (*CHS*), and chalcone isomerase (*CHI*), coumarate-3-hydroxylase (*C3H*) key enzyme gene (Table 3). However, the key enzyme transcriptional activator protein (*ANR*) in the last step of the flavonoid synthesis pathway was not detected in the two species, therefore, it means that the key enzyme genes that affect the synthesis of flavonoids are the upstream genes, which play a major regulatory role. In synthetic ways of antioxidant enzyme, the percent of upregulated genes in invasive species was 35%, while the hybrid was only 9% (Table 4). These differences were consistent with the comparison of flavonoid content.

The differences in the SOD contents in the two species were not obvious, therefore, we hypothesized that under Cd stress, the removal of excessive reactive oxygen in two plants may be more dependent on the POD enzyme in the leaves, so that H_2_O_2_ was directly converted into H_2_O and O_2_. Under Cd stress, the difference in antioxidant capacity is due to the difference in the expression of genes regulating peroxidase. For example, the expression of Cytochrome C peroxidase was up-regulated, which led to the increase of POD enzyme content in the leaves of the two species. Under Cd stress, the higher accumulation of ROS in plant leaves, the more antioxidant enzymes need to be regulated for scavenging. Under the Cd stress, the genes of peroxidase were significantly upregulated in invasive species, so the POD content was higher than in the hybrid.

### Expression of Genes Associated With Hormone Signal Transduction in Two Species Under Cd Stress

**FIGURE 6:**
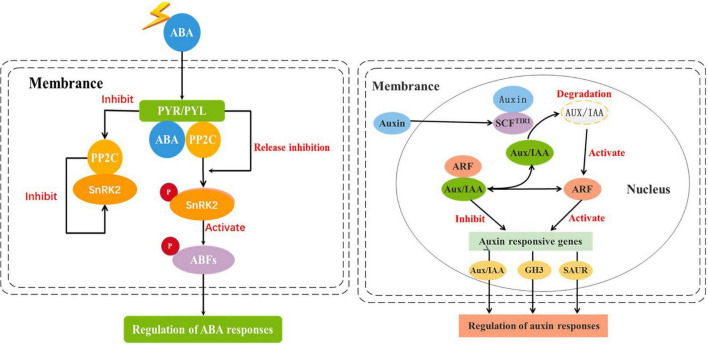
The key genes in abscisic acid ABA **(Left)** and auxin IAA **(Right)** synthesis pathway. The DEGs (Pyrabactin resistance/pyr1-like (PYR/PYL), Serine/threonine protein phosphatase (*PP2C*), Sucrose non-fermenting 1-related protein kinase 2 (*SnRK2*), ABA responsive element binding factors (*ABFs*)) in invasive plant and hybrid under Cd stress were located in the ABA synthesis pathway, and the DEGs (Auxin receptor (*TIR1*), Auxin/indole-3-acetic acid (*Aux/IAA*), Auxin response factor (*ARF*), Gretchen hagen 3 (*GH3*), Small auxin-up RNA (*SAUR*)) were located in the IAA synthesis pathway.

Corresponding to the results of plant hormone (ABA, IAA) in the leaves of two species, the genes related to these hormones also showed up or downregulation. Among these, under Cd stress, the genes associated with ABA synthesis were upregulated, and resulted in 22% in invasive species, while only 6.9% in the hybrid (Table 4). Due to the increase of ABA content in the leaves, the downstream genes in the regulatory pathway of ABA were upregulated, for example, the expression of the first key gene pyrabactin resistance/pyr1-like (*PYR/PYL*) in the signaling pathway was upregulated. Accordingly, this gene can combine with ABA and will restrain the activity of the gene serine/threonine protein phosphatase (*PP2C*), so the downstream gene sucrose non-fermenting 1-related protein kinase 2 (*SnRK2*) in the regulatory pathway was upregulated (Table 3). The phosphorylation of *SnRK2* can phosphorylate ABA responsive element binding factors (*ABFs*), and finally, the ABA signal will be activated (Figure 6 Left).

In contrast to the upregulation of the genes associated with the ABA signal, the expressed genes in the IAA signaling pathway were not consistent in two plants under Cd stress (Table 3). The key genes in the IAA signaling pathway were downregulated in invasive species, while in the hybrid, the genes were upregulated. As to the number of differentially expressed genes enriched to the IAA signaling pathway, there were 4% in the hybrid, which is remarkably different from invasive species (30.4%) (Table 4).

Under Cd stress, the content of IAA in the leaves of the hybrid was higher than that in invasive species. In the pathway of IAA signal transduction, the genes were upregulated in the hybrid under Cd stress, while in invasive species, the pathway of IAA signal transduction was inhibited. More IAA can combine with the protein of auxin receptors (*TIR1*), and then, it will induce the gene auxin/indole-3-acetic acid (*Aux/IAA*) ubiquitination and further degradation, releasing more genes auxin response factor (*ARF*), which promotes the expression of auxin responsive genes. In contrast, if the content of IAA decreased, the transcription factor *Aux/IAA* in the signaling pathway will combine with *ARF* to form a heterodimer, which can inhibit the expression of the auxin response gene, and then limit cell growth (Figure 6 Right).

## Discussion

### Photosynthetic Characteristics and Response Mechanism to Cd Stress in Two Species

Invasive species have a stronger ability in growth and photosynthesis than native relatives, but in some special environments (e.g., seasonal flooding, heavy metals, and high temperature) (Zhang et al., 2020b), native relatives have certain adaptability in the long-term growth and evolution process. Therefore, by crossing with native relatives, invasive species can obtain tolerance in a stressful environment. Studies have shown that the photosynthetic process of many plants is affected by heavy metals (Curado et al., 2010; Dominguez et al., 2011; Augustynowicz et al., 2014). The growth and development of plants will be inhibited under Cd stress, and the photosynthetic efficiency is often damaged most significantly. In our study, under the Cd stress, the key protein genes related to the photosynthesis pathway of invasive species were downregulated, such as photosystem I, photosystem II, cytochrome b6/f complex, electron transport vector, and key protein genes of ATP synthase, but the effects of Cd stress on the hybrid were not obvious in the photosynthetic process. The biomass of the hybrid was higher than invasive species after Cd stress treatment. This indicated that the photosynthetic process of invasive species was disrupted by Cd stress, the hybrid offspring of invasive species hybridizing with native species has a certain tolerance to Cd stress, and it can maintain higher photosynthetic capacity, due to the strong Cd tolerance of native species in our previous studies (Zhang et al., 2020b). Therefore, more genes related to photosynthetic physiology are highly expressed in hybrids under Cd stress, which also proves that invasive species can invade in Cd stress environment by hybridization with native relatives.

### The Response of Antioxidant Enzyme Characteristics of Two Species to Cd Stress

The tolerance of plants to a stressful environment is not only reflected in growth but also in how the metabolic activities in the body resist the stressful environment. The higher the resistance index, the stronger the tolerance of plants. Under Cd stress, plants will accumulate a large amount of ROS, such as O^2–^, H_2_O_2_, and OH^–^, which will damage plants and affect their normal physiological function (Thevenod, 2009). However, plants can activate reactive oxygen scavenging enzymes and produce a series of antioxidant enzymes such as superoxide dismutase (SOD), peroxidase (POD). Among them, the SOD enzyme can convert O^2–^ into H_2_O_2_ and O_2_, while the APX enzyme can further convert H_2_O_2_ into H_2_O, POD enzyme can also convert H_2_O_2_ into H_2_O and O_2_ (Karuppanapandian et al., 2011). Thus, the damage of reactive oxygen to the cell membrane and the destruction of photosynthetic structure can be reduced to ensure the normal growth and development of plants. In this experiment, under Cd stress, the removal of excessive reactive oxygen in two plants may be more dependent on the content of the POD enzyme in the leaves. Compared with hybrid, the stress of Cd on the invasive plant is stronger, so the invasive plant has high POD to resist the damage of Cd stress. Therefore, the genes related to antioxidant capacity are also upregulated. For example, the expression of Cytochrome C peroxidase was upregulated, which led to the increase of POD enzyme content in the leaves of the two species, and similar upregulation results were also found in other plants, such as *Solanum nigrum* and *S. torvum* (Liu et al., 2013).

Non-enzymatic antioxidants are another type of antioxidant system for scavenging ROS, generally including ascorbic acid, glutathione, flavonoids, and phenols. These substances can directly react with ROS to remove them or act as substrates of antioxidant enzymes to remove ROS, and then, reduce or alleviate the damage of adverse conditions to plants (Telesinski et al., 2008; Kumar and Prasad, 2018). Thus, the higher the content of non-enzymatic antioxidants in plants, the greater the stress on plants. In our experiment, under Cd stress, the changes of flavonoids and total phenols were different in the two plants. In invasive plants, the total phenols and flavonoids were higher than those in hybrid, and the key enzyme genes of flavonoid synthetic pathways, such as *CHI* and *C3H*, were also highly expressed. So that, after hybridization between invasive species and native species, the hybrid can tolerate a high Cd environment, and reduce the synthesis of antioxidant metabolites (flavonoids and total phenols). It further showed that invasive plant is not tolerant to Cd, but through hybrid, the tolerance to Cd is greatly improved.

### The Response of Hormones of Two Species to Cd Stress

In the stress environment, the indexes of plant hormone contents and the genes related to regulatory and synthesis can reflect the degree of plant response to the environment. There is a negative correlation between IAA and the degree of stress. The higher the content of IAA, the more the plants can grow in a stressful environment. Under Cd stress, the content of IAA in the leaves of the hybrid was higher than that of invasive species. In the pathway of IAA signal transduction, the genes were upregulated in the hybrid under Cd stress, while in invasive species, the pathway of IAA signal transduction was inhibited. Studies found that more IAA can combine with the protein of *TIR1*, and then, it will induce *Aux/IAA* ubiquitination and further degradation, releasing more *ARF*, which promotes the expression of auxin responsive genes. In contrast, if the content of IAA decreased, the transcription factor *Aux/IAA* in the signaling pathway will combine with *ARF* to form a heterodimer, which can inhibit the expression of the auxin response gene, and then limit cell growth. Therefore, the hybrid can better regulate plant growth under Cd stress than invasive species.

Contrary to IAA, ABA was considered a “stress hormone,” which is a signal substance of plants against biological and abiotic stresses (Pérez Chaca et al., 2014). Studies showed that under Cd stress, exogenously increasing ABA content would lead to an increase in Cd toxicity to rice, thus the leaf growth will be significantly inhibited (Moya et al., 1995). Therefore, the level of plant ABA content also implies the stress degree of Cd to plants. If plants cannot tolerate Cd stress, the content of ABA will increase. In this study, under Cd stress, compared with hybrid, invasive plant has higher ABA content, which also proves that invasive plant is less resistant to Cd stress. In a hybrid, due to the decrease of ABA, the combination with the downstream gene *ABF* was also reduced, which slowed down the senescence of leaves and thereby reduced the inhibition of plant growth and development.

## Conclusion

Through the study of the tolerance to Cd stress of invasive species *S. trilobata* and the hybrid, we found that *S. trilobata* was weaker than the hybrid in the tolerance to Cd stress, and it was proved in photosynthetic parameters, antioxidant enzyme system, and hormone content. Due to industrial pollution and other reasons, the soil of China is seriously polluted by Cd (Liang et al., 2017), while the native species *S. calendulacea* has a stronger ability to withstand Cd stress (Zhang et al., 2020b). The sites which are suitable for *S. calendulacea* to grow, such as swamps, roadsides, or wasteland, are often the places where Cd pollution in the soil is most serious (Zhang et al., 2020b). Therefore, Cd becomes a limiting factor for the further diffusion distribution of *S. trilobata*. *S. trilobata* crossed with native species *S. calendulacea* to form a hybrid, which greatly broke the limit of Cd stress. Although *S. trilobata* can hardly grow in the heavy Cd pollution environment, its hybrid species can diffuse and grow in the high Cd environment. From the genetic point, this will be a new way of gene invasion, that is, the combination with native species, through the hybrid to spread the genes of the invasive species. There are many ways and strategies for the success of plant invasion. Producing hybrids with native relatives is an important way for the successful invasion of invasive species. Hybridization advantage makes hybrids able to adapt to the environment that invasive species cannot adapt to.

This phenomenon is not unique. In fact, some invasive species would produce hybrid species with native species, and then form a new invasive species, such as *Spartina alterniflora* which is widely distributed in China (Xia et al., 2015). Therefore, for the monitoring and management of alien species, the native species of the same genus may become the pusher for alien species to invade new places, and it may realize the successful invasion of a new environment through the generation of hybrid offspring.

## Data Availability Statement

The original contributions presented in the study are publicly available. This data can be found here: NCBI Sequence Read Archive under accession numbers SRR8755022/SRR8755023/SRR8755024/SRR18652248/SRR18652247/SRR18652246 (*Sphagneticola calendulacea*) and SRR8755025/SRR8755020/SRR8755021/SRR18652245/SRR18652244/SRR18652243 (*Sphagneticola trilobata*).

## Author Contributions

CP conceived, designed, supervised this research, and agreed to serve as the author responsible for contact and ensures communication. MC, LZ, QZ, HZ, and XG did the experiments. MC analyzed the data. LG and MC wrote the manuscript. All authors have read and approved the final version of the manuscript.

## Conflict of Interest

The authors declare that the research was conducted in the absence of any commercial or financial relationships that could be construed as a potential conflict of interest.

## Publisher’s Note

All claims expressed in this article are solely those of the authors and do not necessarily represent those of their affiliated organizations, or those of the publisher, the editors and the reviewers. Any product that may be evaluated in this article, or claim that may be made by its manufacturer, is not guaranteed or endorsed by the publisher.
