# Supplementary material for: Adaptation of the Invasive Plant (Sphagneticola trilobata L. Pruski) to a High Cadmium Environment by Hybridizing With Native Relatives
Source: Front Plant Sci. 2022 Jun 28;13:905577. doi: 10.3389/fpls.2022.905577 (PMC9277564; doi:10.3389/fpls.2022.905577)
Supplement: Supplementary file 1 [file Table_1.docx]

**Supplementary material**

**Table 1.** Enrichment analysis by KEGG on the pathways of differentially expressed genes (DEGs) in the leaves of two plants under cadmium stress

| Pathway | DEGs amount(percent) | P | Q |
| --- | --- | --- | --- |
| **Invasive species (*S. trilobata*)** | 3789 |  |  |
| Photosynthesis - antenna proteins | 143(3.77%) | 0 | 0 |
| Photosynthesis | 162(4.28%) | 0 | 0 |
| Phenylpropanoid biosynthesis | 195(5.15%) | 0 | 0 |
| Porphyrin and chlorophyll metabolism | 81 (2.14%) | 0 | 0 |
| Biosynthesis of unsaturated fatty acids | 89 (2.35%) | 0 | 0 |
| Stilbenoid, diarylheptanoid and gingerol biosynthesis | 65 (1.72%) | 0 | 0 |
| Flavone and flavonol biosynthesis | 28 (0.74%) | 0 | 0 |
| Fatty acid metabolism | 143(3.77%) | 0 | 0 |
| Zeatin biosynthesis | 43 (1.13%) | 0 | 0 |
| Plant hormone signal transduction | 230(6.07%) | 0 | 0 |
| **Hybrid** | 1250 |  |  |
| Photosynthesis - antenna proteins | 87 (6.96%) | 0 | 0 |
| Photosynthesis | 90 (7.2%) | 0 | 0 |
| Porphyrin and chlorophyll metabolism | 53 (4.24%) | 0 | 0 |
| Biosynthesis of unsaturated fatty acids | 41 (3.28%) | 0 | 0 |
| Fatty acid metabolism | 57 (4.56%) | 0 | 1.00E-06 |
| Nitrogen metabolism | 29 (2.32%) | 0 | 4.00E-06 |
| Phenylpropanoid biosynthesis | 54 (4.32%) | 5.20E-05 | 9.14E-04 |
| Steroid biosynthesis | 20 (1.6%) | 7.40E-05 | 1.14E-03 |
| Glyoxylate and dicarboxylate metabolism | 48 (3.84%) | 1.44E-03 | 1.97E-02 |
| Plant hormone signal transduction | 80 (6.4%) | 4.39E-03 | 5.40E-02 |
